# Myotube elasticity of an amyotrophic lateral sclerosis mouse model

**DOI:** 10.1038/s41598-018-24027-5

**Published:** 2018-04-12

**Authors:** Béla Varga, Marta Martin-Fernandez, Cécile Hilaire, Ana Sanchez-Vicente, Julie Areias, Céline Salsac, Frédéric J. G. Cuisinier, Cédric Raoul, Frédérique Scamps, Csilla Gergely

**Affiliations:** 1 L2C, Univ Montpellier, CNRS, Montpellier, France; 2 INM, INSERM, Univ Montpellier, Montpellier, France; 3 LBN, Univ Montpellier, Montpellier, France

## Abstract

Amyotrophic lateral sclerosis (ALS) is a fatal neurodegenerative disease that affects the motor system leading to generalized paralysis and death of patients. The understanding of early pathogenic mechanisms will help to define early diagnostics criteria that will eventually provide basis for efficient therapeutics. Early symptoms of ALS usually include muscle weakness or stiffness. Therefore, mechanical response of differentiated myotubes from primary cultures of mice, expressing the ALS-causing *SOD1*^*G93A*^ mutation, was examined by atomic force microscopy. Simultaneous acquisition of topography and cell elasticity of ALS myotubes was performed by force mapping method, compared with healthy myotubes and supplemented with immunofluorescence and qRT-PCR studies. Wild type myotubes reveal a significant difference in elasticity between a narrow and a wide population, consistent with maturation occurring with higher actin expression relative to myosin together with larger myotube width. However, this is not true for *SOD1*^*G93A*^ expressing myotubes, where a significant shift of thin population towards higher elastic modulus values was observed. We provide evidence that SOD1 mutant induces structural changes that occurs very early in muscle development and well before symptomatic stage of the disease. These findings could significantly contribute to the understanding of the role of skeletal muscle in ALS pathogenesis.

## Introduction

Amyotrophic lateral sclerosis (ALS) is a neurodegenerative disease, which causes a gradual degradation of motor functions, with an incidence of 2.16 per 100 000 person-years in Europe^1^. The scientific interest to investigate the disease started to raise in the 90’s, following the discovery of ALS-causing mutations in the Cu/Zn superoxide dismutase (SOD1) gene and new insights in the glutamate neurotransmitter system^2^. The origin of ALS in 5–10% of the cases is familial, while the rest of the patients diagnosed are considered as sporadic^2^. The survival time after first symptoms for 50% of the patients is below 30 months, while only 20% of patients survive after 5 years and a small percentage are alive after 10 years^3^. Mutations in SOD1 are responsible for approximately 20% of the familial and 5% of the seemingly sporadic ALS^2,4,5^. Transgenic mice overexpressing human mutated SOD1 gene provided a robust model mimicking the main pathological traits of human ALS^5^.

Nanobiomechanics, as an emerging powerful technology to explore mechanical aspects of biological matter at the nanoscale, has recently opened new horizons by generating a significant contribution in the comprehension of various human diseases. Besides helping in the understanding of mechanisms behind disease progression, biomechanical investigation of physiological and pathological processes of different diseases provided valuable knowledge for the development of therapies^6^.

The main tools of nanobiomechanics are atomic force microscopy (AFM)^7,8^, optical tweezer/stretcher^9,10^ and cell traction force microscopy^11^, but other techniques have also been used to study single cell mechanical properties such as magnetic twisting cytometry^12^, micropipette aspiration^13^, cell poker^14^ or scanning acoustic microscopy^15^. AFM, besides recording high-resolution three-dimensional images on biological samples in native physiological environments, holds the advantage to easily manipulate the sample, with forces at pico-newton scale. Thanks to this unique feature, AFM has the potential to spatially resolve the sample’s elasticity by nanoindentation, and to map sample properties that are directly correlated to the topography.

Some early AFM studies on skeletal muscles have investigated the surface morphology and transverse elasticity of rabbit and drosophila myofibrils^16,17^. The myofibrils sectioned from mature skeletal muscle have shown elasticity values from 11 to 94 kPa, depending on the loci. Mathur *et al*. performed the first measurements on intact cells. They compared general elasticity of skeletal and cardiac muscle cells in liquid, getting 24.7 ± 3.5 kPa and 100.3 ± 10.7 kPa elastic modulus values respectively^18^. A work from the same group investigated elastic modulus of skeletal muscle cells throughout differentiation^19^, and have reported a sharp increase in the average elasticity from 10 kPa (day 1) to 35–45 kPa after 8 days *in vitro* (DIV) differentiation. In both studies, C2C12 murine myoblast cell line was used. The first complete three-dimensional topography and mechanical characterization of intact, living skeletal muscle fibers were performed by Defranchi and his coworkers^20^, measuring an average elasticity value of 61 ± 5 kPa on the sarcolemma of the fibers, while Ogneva *et al*. have characterized mechanical properties of muscle fibers at sites corresponding to Z-disks, M-bands, and regions between them^21^.

Although several studies examined mechanical properties of healthy skeletal muscle cells from different origin and in various states of differentiation, few investigated the effect of diseases on skeletal muscle cell elasticity, the majority of which addresses mainly muscular dystrophies^22–24^. To the best of our knowledge there is no study addressing the mechanical properties of developing ALS diseased satellite cells. Here, our objective was to study the elasticity of ALS myoblasts and myotubes isolated from the muscles of asymptomatic *SOD1*^*G93A*^ mice to understand whether early structural disturbances could contribute to ALS pathogenesis, which may lead to advancements in early diagnosis and therapeutics of ALS.

## Results

### Elasticity of myoblasts in stage of elongation

**Figure 1:**
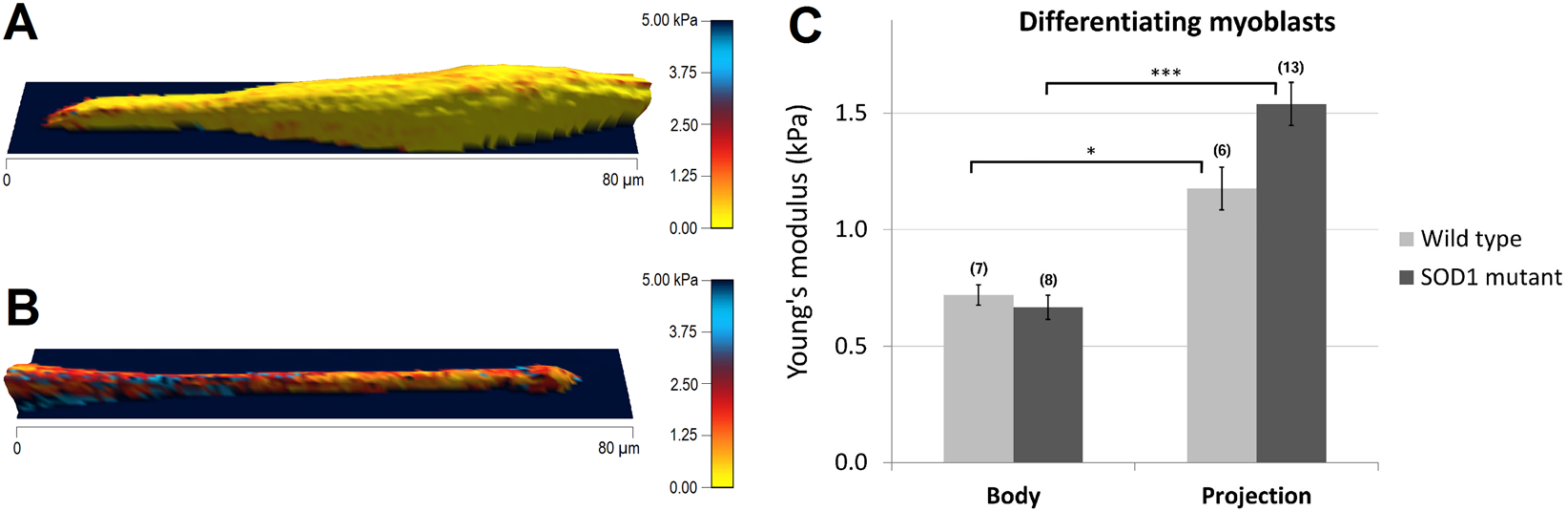
Elasticity of wildtype and SOD1 mutant myoblast initiating elongation. 3D-reconstructions of differentiating myoblasts with elasticity coloration are represented in different stages: a spindle-like morphology (**A**) and an elongated projection (**B**). Yellow color depicts softer portions, while red to blue colors show stiffer regions. Color bar goes from 0 kPa (yellow) to 5 kPa (dark blue). (**C**) Comparison of cell body’s and projection’s average elasticity between wildtype and SOD1 mutant single myoblasts, prior cell fusion (*p < 0.05, ***p < 0.001, Mann-Whitney test). The number of the analyzed force maps is indicated in brackets.

To obtain comprehensive information about the elastic properties, AFM was used and whole cell force maps recorded on primary myoblasts after keeping them in differentiation medium from 6 to 8 DIV. The heterogeneity of cell culture allows examining not only different populations of myotubes, but single myoblasts as well. Figure 1 displays the elastic modulus distribution along the surface of myoblasts in two different stages of myotube formation. Figure 1A depicts a myoblast in spindle like morphology stage with reduced and homogenously distributed elasticity, while in Fig. 1B the projection of a more elongated, but still single, myoblast is represented. The average elastic modulus values measured over the central area of the cells were similar for wildtype and SOD1 mutant myoblasts, amounting 720.47 ± 88.55 Pa, *n* = 7 and 667.25 ± 103.72 Pa, *n* = 8, respectively (Fig. 1C-Body). Elastic modulus of projections of the more elongated myoblasts were significantly higher compared with body values, amounting 1176.64 ± 183.94 Pa (*n* = 8, * *p* < 0.05) in wildtype myoblast and 1540 ± 184.50 Pa (*n* = 13, *** *p* < 0.001) in *SOD1*^*G93A*^ myoblasts. As also observed for central area, no differences in projections were observed between the two genotypes (Fig. 1C-Projection).

**Figure 2:**
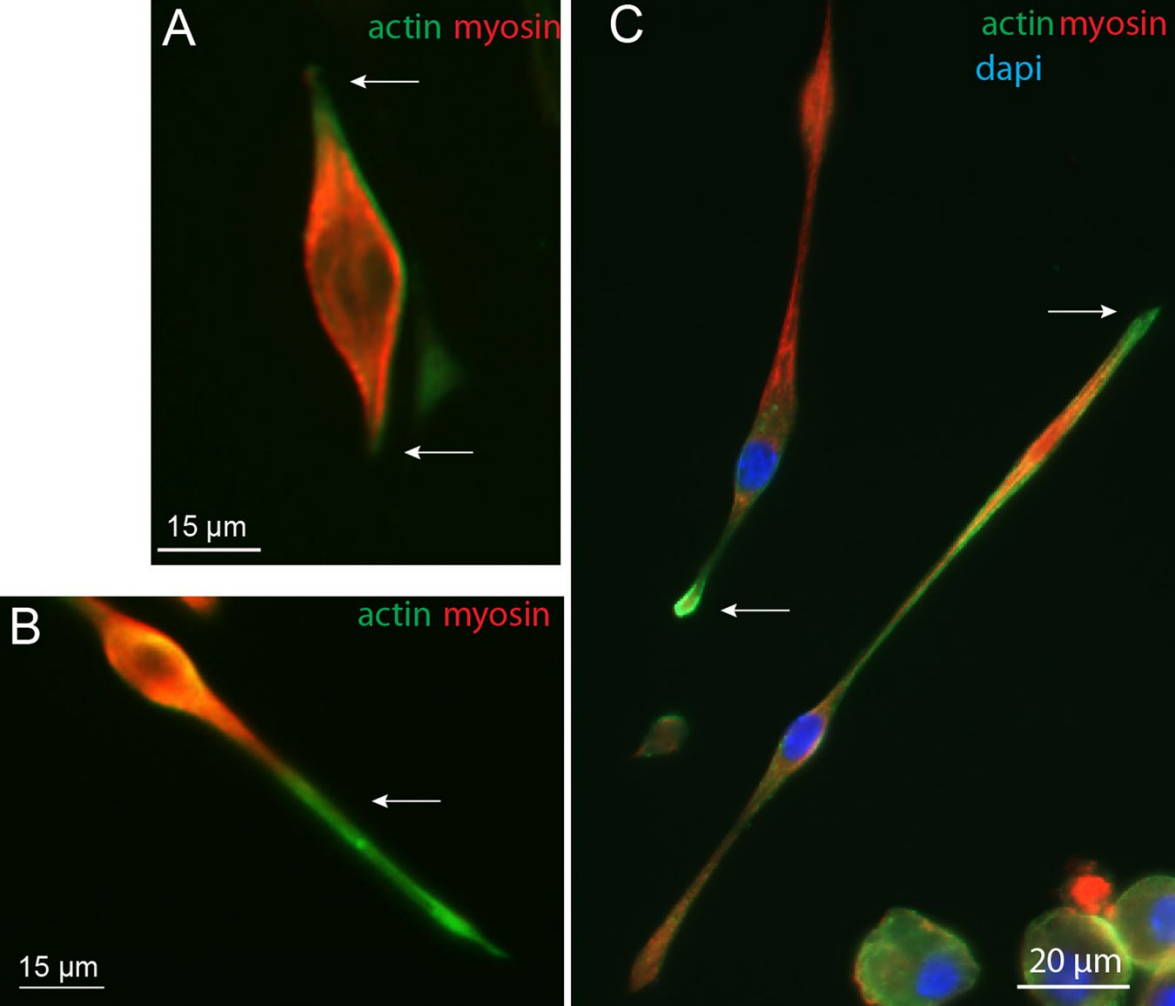
Immunofluorescence of actin and myosin. Immunofluorescence images with actin (green), myosin (red) and nucleus (blue) staining of myoblasts in the process of elongation, observed at 7 DIV.

Previous experiments reported that elastic modulus changes during myotube maturation is mainly dependent on actin and myosin, but not on beta-tubulin levels^19,25^. To correlate the observed elasticity changes between soma and projection with cytoskeletal protein content, the ratio of actin-myosin expression was determined using immunocytochemistry. As observed in earlier studies, myoblasts *in vitro* have bipolar-shaped forms prior fusion (Fig. 2) with actin cytoskeleton playing a major role in this differentiation process^26^. Double staining experiments clearly show that myosin expression was higher than actin in the soma, while the opposite was observed in projections (Fig. 2A,B arrows). Unlike myosin, actin staining was expressed up to the extremities of the bundles during the process of myoblast elongation which is consistent with its structural role for initiating and maintaining the structure of growing processes^27,28^ (Fig. 2C).

Altogether, these results suggest that low Young’s modulus values (around 500 Pa) reflect myosin abundance while high values (from 1000 Pa) would be a marker of higher actin expression over myosin. In addition, as no differences in elasticity were evidenced between wildtype and *SOD1*^*G93A*^ myoblasts, this suggests similar expression and compartmentation in these cytoskeletal proteins at this early stage.

### SOD1 mutant myotubes show increased elastic modulus

**Figure 3:**
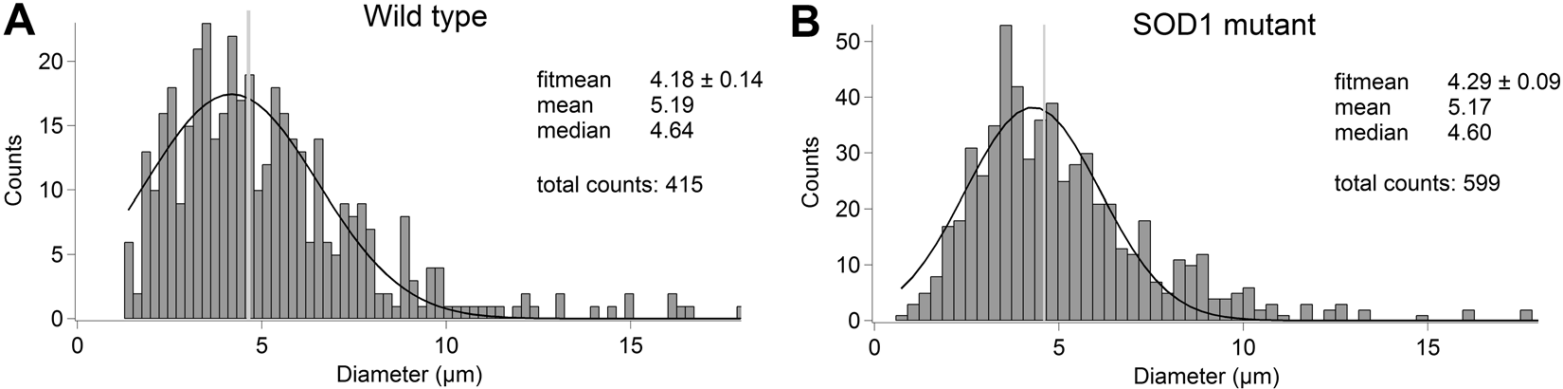
Transverse dimension distributions. (**A** and **B)** Shows the thickness distributions, measured on optical images, of wildtype and SOD1 mutant myotubes, respectively. Black curves represent the Gaussian fitting, while vertical gray lines indicate the median of the data.

As cell cultures present large heterogeneity regarding the diameter of myotubes during fusion process, we performed a Gaussian distribution analysis of myotube thickness as depicted in Fig. 3. Tube diameter varies from 1.7 µm to 15 µm, with a mean value of 5.19 ± 0.14 µm (*n* = 415) for wildtype and 5.17 ± 0.11 µm (*n* = 599) for SOD1 mutant myotubes (p = 0.89, t-test). The absence of significant differences between wildtype and SOD1 mutant myotube diameter suggests that at this stage, the mutation does not induce atrophy. Therefore, we used the median value of 4.62 µm, as a cut-off value for a narrow/wide classification, due to an equal grouping of data into thin and thick populations.

**Figure 4:**
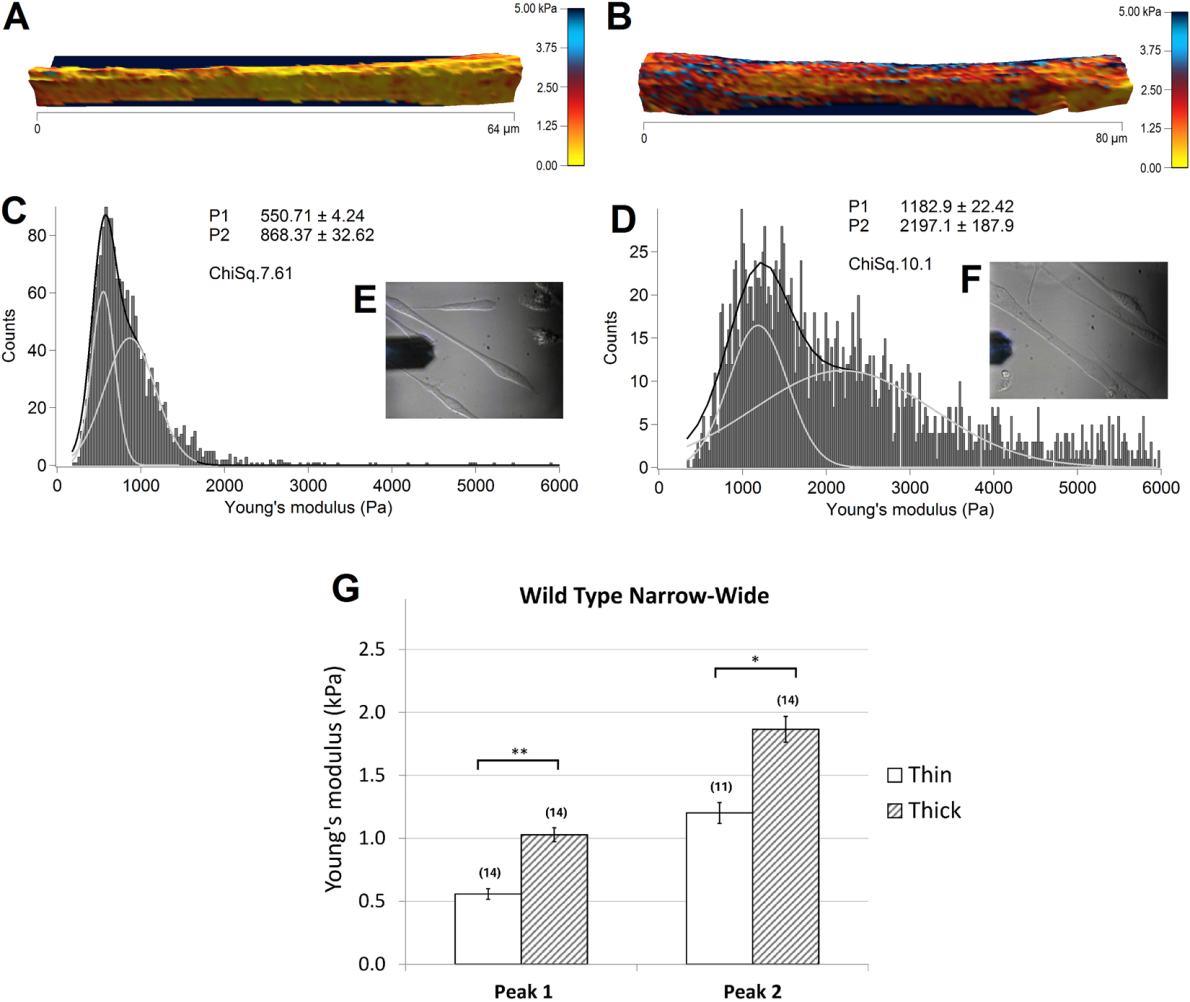
Elasticity of thin (left) and thick (right) wildtype myotubes. 3D-reconstruction of representative thin (**A**) and thick (**B**) myotubes, with elasticity coloration. Yellow color shows softer portions, while red to blue colors show stiffer regions. Color bar goes from 0 kPa (yellow) to 5 kPa (dark blue). (**C** and **D**) represent the elasticity distributions of force maps corresponding to A and B respectively. Light gray curves are single Gaussian functions, while black curves represent the sum of the two. Insets (**E** and **F**) show the optical images of the measured myotubes. (**G**) The bars represent the obtained average of the double Gaussians (Peak 1 and Peak 2), fitted on the elasticity value distributions of individual force maps, measured on thin and thick myotubes. In brackets the number of the analyzed force maps is denoted. Error bars are standard error of the mean. As indicated, significant difference was found on both peaks of the Gaussian fit (*p < 0.05, **p < 0.01, Mann-Whitney test).

**Figure 5:**
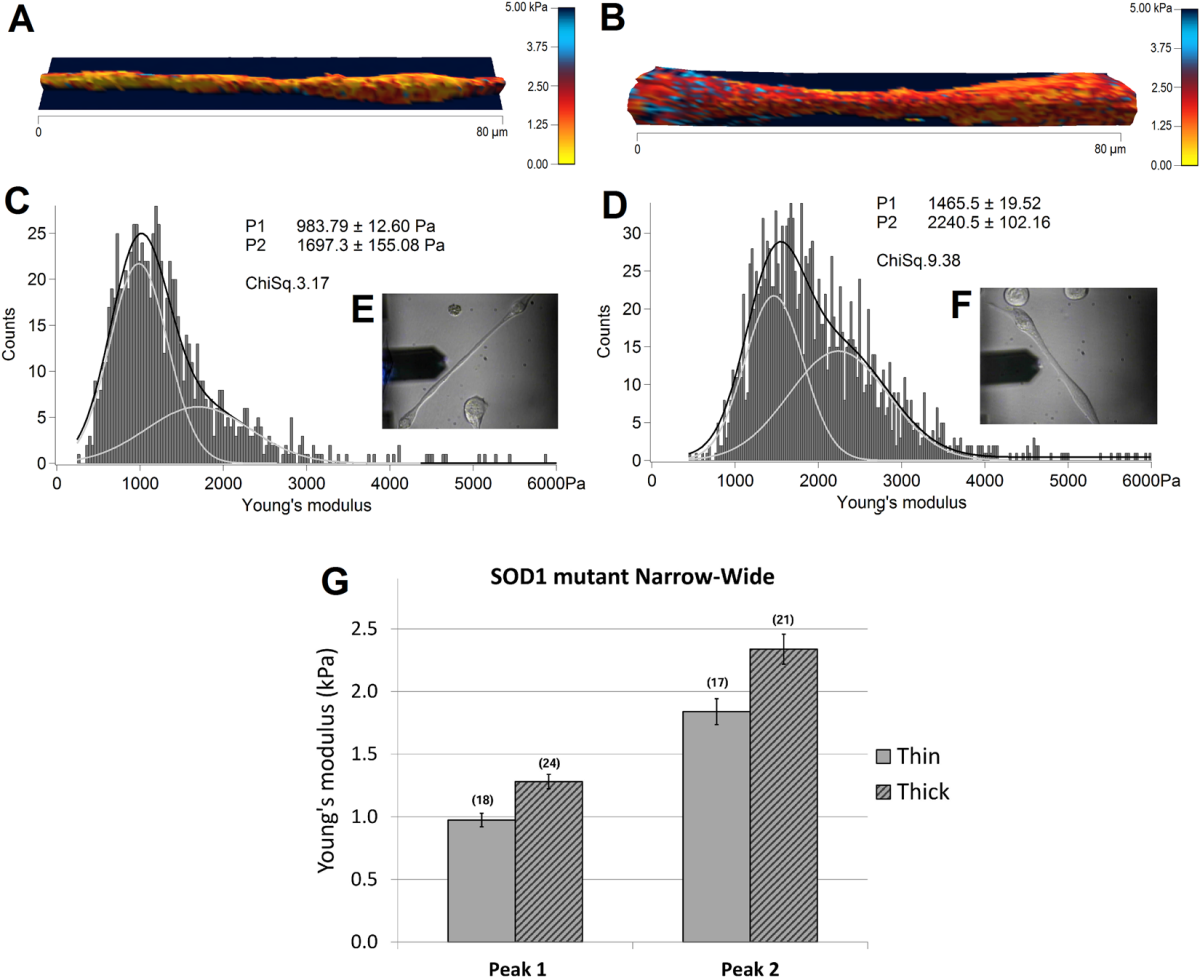
Elasticity of thin (left) and thick (right) SOD1 mutant myotubes. 3D-reconstructions with elasticity coloration of thin (**A**) and thick (**B**) SOD1 mutant myotubes are presented. Yellow color indicates softer portions, while red to blue colors show harder regions. Color bar goes from 0 kPa (yellow) to 5 kPa (dark blue). (**C** and **D**) represent elasticity distributions of force maps A and B respectively. Light gray curves are single Gaussians, while black curves are the sum of the two. The insets (**E** and **F**) show optical images of the measured myotubes. (**G**) The bars represent the obtained average of the double Gaussians (Peak 1 and Peak 2), fitted on the elasticity value distributions of individual force maps. In brackets the number of the analyzed force maps is indicated. Error bars are standard error of the mean. No significant difference was found between the two populations.

In the following experiments, AFM was used to compare the elastic modulus of thin and thick wildtype (*n* = 28) and *SOD1*^*G93A*^ (*n* = 42) myotubes (Figs 4 and 5, respectively) from 3 wildtype and 3 *SOD1*^*G93A*^ primary cultures. Figure 4A and B shows two representative topographical images of thin and thick wildtype myotubes, reconstructed from force maps by using the contact point of the collected force curves as the height, and the elasticity parameter for coloration values. The range values of measured elastic modulus had large variations from some hundreds of Pa up to 10 kPa observed not only between individuals, but also within single force maps. These variations were addressed by fitting elasticity distributions of each force map with a sum of two Gaussian functions, illustrated as light gray curves on Fig. 4C,D. Consequently, all force maps were associated with a double average, corresponding to the peaks of the double Gaussian fit, which gives an index of elasticity variability.

Collecting optical images (Fig. 4E,F), before, during and after the measurements, ensured the exclusion of those data where potential morphological changes throughout force volume acquisition were observed. The obtained average elasticity values of double fitting were 557.05 ± 83.81 Pa and 1200.70 ± 165.89 Pa, *n* = 14 for thin myotubes and 1027.88 ± 110.38 Pa and 1865.47 ± 204.14 Pa, *n* = 14 for thick myotubes, showing a significant difference (**p < *0.01*, *p < *0.05*) between the two population on both peaks (Fig. 4G). Therefore, there was an overall significant increase in elasticity of thick myotubes.

Representative elasticity colored topographical maps (A and B) and the corresponding optical images (E and F) of thin and thick *SOD1*^*G93A*^ mutant myotubes are shown in Fig. 5. Force map analysis show Young’s moduli of 974.14 ± 107.34 Pa / 1839.34 ± 206.48 Pa for thin myotubes (Fig. 5C) and 1280.81 ± 115.97 Pa / 2337.47 ± 239.14 Pa for the thick population (Fig. 5D). Interestingly, these values presented no statistical difference suggesting no differences in elasticity between thin and thick *SOD1*^*G93A*^ myotubes (Fig. 5G).

**Figure 6:**
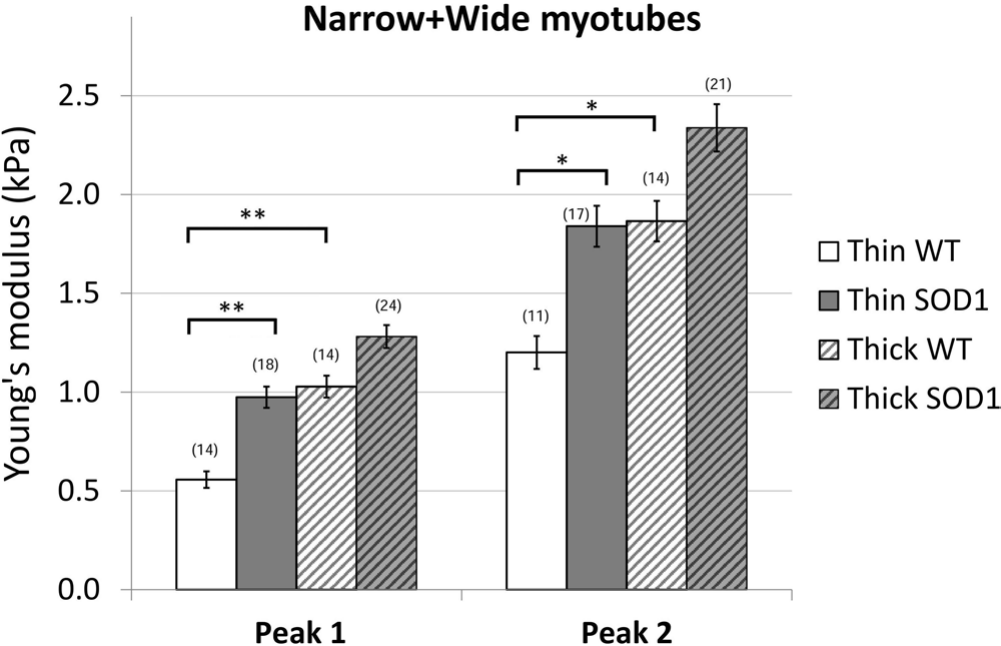
Average elasticity of the wildtype and SOD1 mutant myotubes. The bars represent the obtained average of the double Gaussians (Peak 1 and Peak 2), fitted on the elasticity value distributions of individual force maps. In brackets the number of the analyzed force maps is indicated. Error bars are standard error of the mean. As indicated, the elasticity of thin wildtype myotubes is significantly lower than thin SOD1 and thick wildtype as well (*p < 0.05, **p < 0.01, Mann-Whitney test).

Analysis of elasticity between wildtype and *SOD1*^*G93A*^ myotubes evidenced a shift of the thin myotubes expressing SOD1 mutant towards higher elasticity values compared to wildtype, while there was no significant difference in elasticity values between the two thick populations of wildtype and *SOD1*^*G93A*^ myotubes (Fig. 6). Altogether, these results suggest that the population of wildtype myotubes at 6–8 DIV is highly heterogeneous in term of elasticity, while expression of *SOD1*^*G93A*^ leads to homogenization of the entire population towards hardest structures.

### SOD1 mutation decreases myosin heavy chain gene isoforms expression

From our data on myoblasts, it appeared that a higher content of myosin relative to actin soften the tissue and that wildtype myotubes are softer than *SOD1*^*G93A*^ myotubes. Therefore, we tentatively correlated biophysical properties of myotubes with myosin and actin expression during myotube formation.

**Figure 7:**
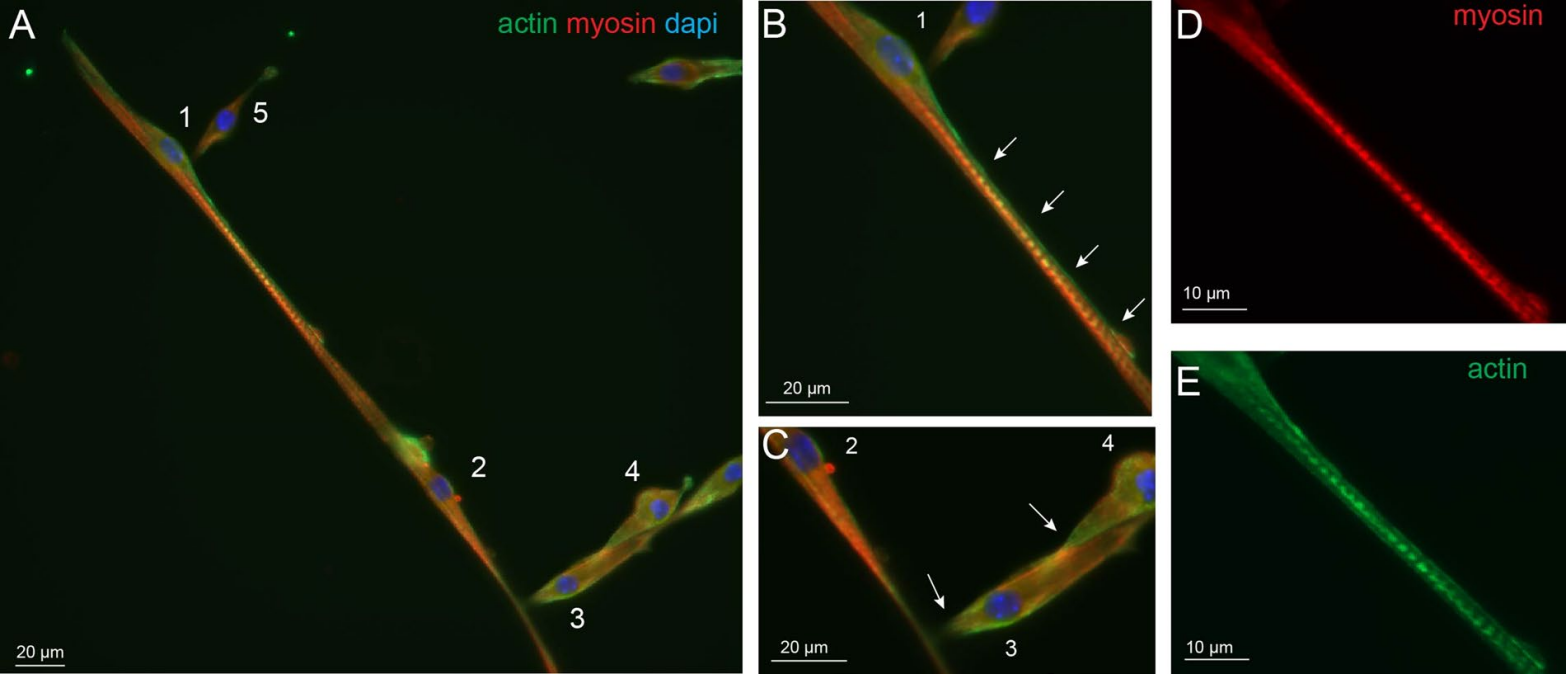
Primary fusion of wildtype myotubes. Immunofluorescence images are presented with actin (green), myosin (red) and nucleus (blue) staining of wildtype myotubes at 7 DIV. (**A**) Primary fusion process can be observed while the actin rich projection of myoblast 1 (**B**, white arrows) is elongated along myotube 2 and seems to initiate fusion with it. Myoblasts 3–5 present fusion initiation with perpendicular orientation on the myotube by an actin rich contact region (**C**, white arrows). The enlargements (**D** and **E**) show the well separated striations of myotube 2 with myosin and actin staining, respectively.

Immunostaining of actin and myosin were performed on wildtype myotubes at 7 DIV in differentiation medium. Using these cytoskeletal markers, two processes of myotube formation could be evidenced. The first correspond to the so-called primary fusion that consists of the construction of a poly-nucleated single fiber as shown in Fig. 7A. In this representative example, myoblast 1 is in the process of fusion that allows seeing an elongated actin structure that seems to initiate the fusion process in the myotube 2. Perpendicular to the fiber, several myoblasts (3–5) make contact together and with the forming myotube using an actin edge before the fusion. This thin myotube illustrates the mixed contribution of actin and myosin with stronger staining for myosin to the fiber that could account for elasticity corresponding to peak 1. In addition, punctiform actin and myosin staining, reminiscent to striation, were observed in some regions of the myotubes (Fig. 7D,E). These spots of actin could account for the harder structures evidenced with peak 2 analysis.

An illustration of thick myotubes is shown in Figure S1 under a fusion process. While we could discern spots of actin, it was much harder to have such resolution with myosin. This seems to corroborate the trends towards harder elasticity in thick myotubes. However, same analysis of actin-myosin staining in *SOD1*^*G93A*^ myotubes did not allow revealing differences compared to wildtype (data not shown).

**Figure 8:**
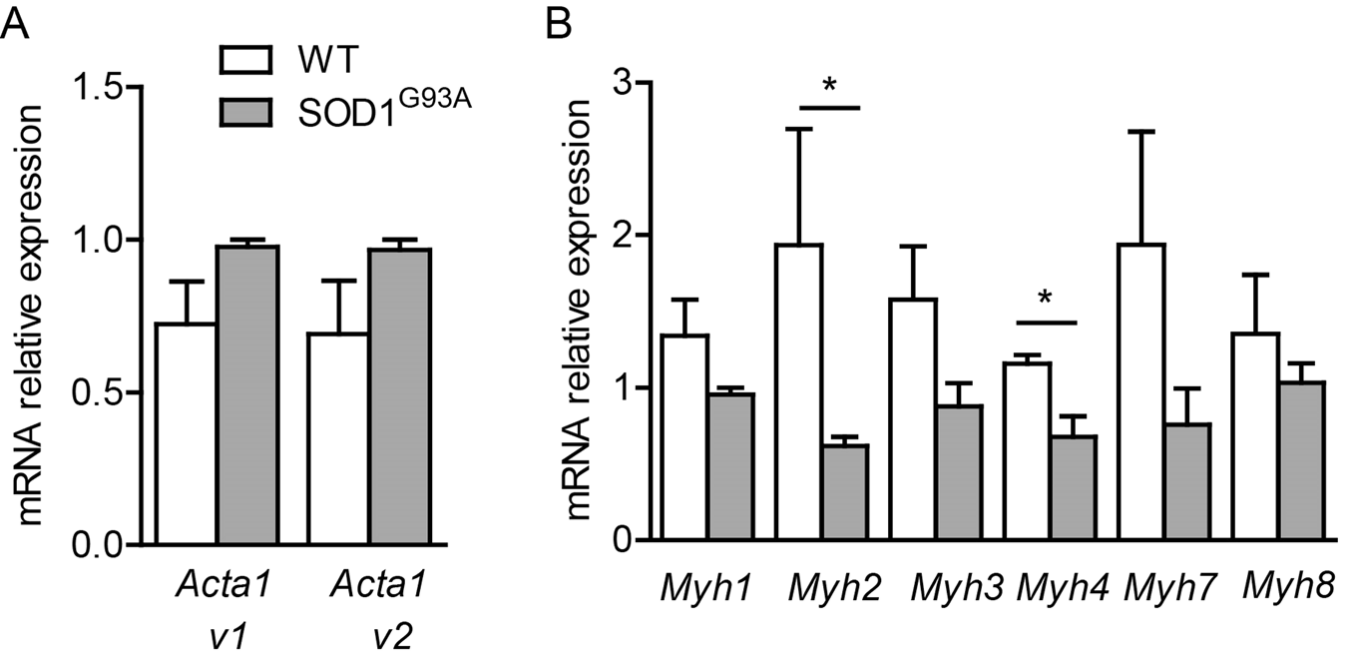
Actin and myosin heavy chain gene expression of wildtype and SOD1 mutant myotubes. Quantitative RT-PCR measurement of (**A**) two actin isoforms (*Acta1 v1*, *Acta1 v2*) as well as (**B**) embryonic (*Myh3*), neonatal (*Myh8*) and adult myosin isoforms *Myh1*, *2*, *4* and *7* expressed in wildtype and SOD1 mutant myotubes (Mann-Whitney test, *p < 0.05; *n* = 3 SOD1^G93A^ and wild-type cultures).

Due to the limit of detection and quantification, we used quantitative RT-PCR to address whether SOD1 mutation induces changes in actin and myosin gene expression in 7 DIV. As displayed in Fig. 8A, the two actin skeletal isoforms, *Acta1 v1* and *Acta1 v*2, tended to be increased in *SOD1*^*G93A*^ expressing myotubes. At this early developmental stage, embryonic and neonatal myosin heavy chain (MHC) genes *Mhy3* and *Myh8* were abundantly expressed and SOD1 mutation induced an apparent decreased expression of *Myh3*, although not significant. Although less expressed than immature isoforms, the adult MHC gene isoforms, *Myh1* coding for MHCIIx, fast fibers, *Myh2* coding for MHCIIa, the fast fatigue resistant fibers, *Myh*4 coding for MHCIIb, the fast fatigable fibers and *Myh*7 coding for MHC-β, the slow fibers^29^, were also detected in 7 DIV myotube cultures (Fig. 8B). Interestingly, among these 4 isoforms, there was a three-fold decrease in *Myh2* expression level and a 2-fold decrease in *Mhy4* expression level in *SOD1*^*G93A*^ myotubes. The overexpression of human *SOD1*^*G93A*^ in myotube was confirmed using primer against the human SOD1 (Ct was around 18, *n* = 3 *SOD1*^*G93A*^ mice, a value reflecting a high expression of the transgene) that was absent in wildtype myotubes (Ct not detectable, *n* = 3 wild type mice).

## Discussion

Elasticity depends both on protein expression and cytoskeletal organization, making nanobiomechanics an effective tool to monitor subtle modifications in diseased cells. As primary myoblasts allow deciphering molecular processes involved in muscle development, aging and repair, we applied atomic force spectroscopy on primary myoblasts isolated from an ALS mouse model to reveal elasticity features during early differentiation stage into myotubes. Although numerous studies were performed on differentiating myotubes and fully differentiated myofibers, most of them have used single force measurements at only specific parts of the cells^19,21^. As cells are largely heterogeneous, mapping the nanomechanical properties of the entire cell is needed providing more comprehensive data to a better understanding of the underlying mechanisms. Accordingly, we performed force maps along large portions of the two types of myotubes, with a resolution of 100 × 20 pixels, where each pixel corresponds to a single force curve. This method allows interpreting results as a three dimensional topographic image with a true elasticity coloration, where the height values are derived from the contact point and the elasticity values from fitted indentation of the force curves.

The process of myotube formation is composed by a series of complex morphological events. It starts with the anchorage and proliferation of myoblasts, which then aligned in a spindle like morphology with growing elongated projections, are constantly searching for other cells and finally followed by cell fusion in case of a successful encounter. Following 6 to 8 days of differentiation cell culture presents a high heterogeneity, which is not limited to myotubes with different thicknesses, but contains a considerable amount of differentiating single myoblasts, still in search of other cells to fuse with. For myoblasts two different states can be also observed. One has usually a spindle like morphology showing generally a homogeneous distribution and rather low values of elasticity, while the second state is characterized by well elongated projections on one or both sides of the spindle like cell body, with an increased elasticity as shown in Fig. 1. Interestingly, while the elasticity of less elongated myoblasts and the central portions of more elongated myoblasts have similar values for both cell types, increased elastic modulus values can be observed for the projections of elongated SOD1 mutant myoblasts. Unlike myoblasts, the elastic inhomogeneity of the myotubes observable in the colored figures can be recognized on the elasticity distributions as a widening effect. To better interpret this inhomogeneity, the histograms of the calculated Young’s modulus values of each force maps were fitted by a sum of two Gaussians (Figs 4C,D and 5C,D).

Elastic modulus was monitored relative to myotube thickness. Width analysis of myotubes shows a continuum from very thin ones, presumably right after their fusion, to rather thick myotubes apparently in a more mature state when even bundles could be observed. This interpretation was corroborated with morphological analysis of the actin and myosin content. The obtained most commonly occurring elastic modulus values were ranged from some hundreds of Pa up to 4 kPa, which is slightly smaller than it would be expected according to previously reported experiments^18,19^. One reason for this could be the use of a C2C12 murine cell line, in the case of these earlier studies, while we used primary murine cells. Another explanation might be the hardness of the collagen coating we used to promote strong anchorage of the cells onto the plate. Indeed, the measured 100 kPa elasticity of the collagen film is far from the physiological elasticity of the extracellular matrix^30^.

Not only the diameter, but also the number of fused cells, as the building blocks of myotubes was highly variable. Previous studies reported a large increase in elastic moduli value of myotubes throughout differentiation^19^. Consistent with these studies, we show that elasticity variations between the different morphologies of wildtype myotubes correlate a higher elastic modulus with maturation state. In SOD1 mutant, elasticity shift towards hardest values in the thin population could suggest that there is an increased maturation process. However, the dimension frequency measurements, using optical microscopy images, show no differences with the wildtype population that argues against an increased number of fusioning ALS myotubes. This result demonstrates that, at this developmental stage, the mutation does not induce atrophy.

No obvious differences were observed between wildtype and *SOD1*^*G93A*^-expressing myotubes using actin and myosin staining. However, the more sensitive quantitative analysis of gene expression evidenced a decrease in MHC genes coding for the fast fatigue resistant fibers (*Mhy2*) and the fast fatigable fibers (*Mhy4*) together with the tendency towards less embryonic myosin expression which could account for the shift of mutant myotubes towards harder elasticity. These results suggest that in addition to motoneuron death, loss of regenerative potential of the ALS-sensitive fiber types contributes to muscle wasting in ALS disease progression. In agreement with our data, several studies on satellite cells obtained at later stage of the same ALS mouse model or from symptomatic ALS patients skeletal muscles evidence decrease in myosin heavy chain protein^31^, altered capacity of satellite cells to activate the myogenic program necessary for muscle mass maintenance^32,33^. Altogether, these data support that the decrease in *Mhy2* and *Mhy4* expression level is a potential contributor to pathology. Moreover, the existence of some threshold level of expression of multiple proteins that contributes to change in membrane elasticity has been also suggested^19^. Therefore, our data obtained at early pre-symptomatic stage point that early defects in myosin heavy chain composition or distribution reflected by changes in elasticity could be a major factor leading to progressive muscular weakness, independently of denervation^34^.

In summary, our force spectroscopy data reveal differences between the nanomechanical behavior of SOD1 mutant myotube populations and wildtype myotubes, consistent with the trend towards a higher actin content and a lower myosin content observed with qPCR experiments in SOD1 mutant myotubes. These results suggest a faster hardening without accompanying faster maturation process of ALS diseased skeletal myoblasts during myotube differentiation.

## Materials and Methods

### Animals

B6.Cg-Tg(SOD1*G93A)1Gur/J (*SOD1*^*G93A*^) mice were maintained on a C57BL/6 background and purchased from Jackson Labs. All experiments were approved by the Direction Départementale des Services Vétérinaires de l’Hérault (Certificate of Animal Experimentation n° B34–65, 19 August 2010), and were done in compliance with the European community and national directives for the care and use of laboratory animals.

### Myoblast isolation and differentiation

For primary culture of satellite cells, we used the offspring of female C57BL/6 and male *SOD1*^*G93A*^ mice. Satellite cells were obtained from hind limb muscle of 3–4 weeks old mice (pre-symptomatic for *SOD1*^*G93A*^). Two females and one male were used for wild-type primary cultures and three females for *SOD1*^*G93A*^ mice. After mincing muscles, an enzymatic digestion was performed in Ham’s F10 media (Gibco) supplemented with 2.5 mM CaCl_2_, 0.5 mg/ml dispase II (Sigma) and 10 mg/ml collagenase B (Sigma). Following 15 minutes incubation twice at 37 °C, a mechanical dissociation was performed. Muscle digest was passed through a 70 µm mesh filter, transferred to a 15 ml tube and spinned 5 min at 1000 rpm. The pellet was diluted in culture media (Ham’s F10, 20% fetal bovine serum, 2% penicillin/streptomycin, 2.5 ng/ml recombinant human fibroblast growth factor-basic, bFGF; Gibco) and placed in a 60 mm uncoated plastic plate for 1 hr to favor fibroblasts adherence. Afterwards, the content was removed and placed into a plate coated with net-like patterned structure of collagen (as described in details elsewhere^28^) at 37 °C, 7% CO_2_. Satellite cells were allowed to multiply until they reach 70–80% confluence and then split with 0.25% trypsin to enhance the colony. Experiments were performed from fourth to fifth passage. For myoblasts differentiation and fusion, the serum was decreased to 2%.

### Atomic force microscopy

The experimental system was an Asylum Research MFP-3D atomic force microscope mounted on an Olympus ix-71 inverted optical microscope, used both for optical imaging and force spectroscopy.

For all measurements gold-coated silicon-nitride Bio-levers (BL-RC150VB, Olympus, Japan) were used, having a nominal spring constant of 30 pN/nm and a resonant frequency of 37 kHz in air, which drops to 6 kHz in liquid. The cantilevers were equipped with a V-shaped tip, having a half-opening angle of 45° and a radius around 30 nm. After 6 to 8 days of differentiation *in vitro*, the myotubes were transferred under the AFM head in serum free Leibovitz medium (Sigma), which enables maintaining the physiological conditions for long time in CO_2_ free atmosphere. The measurements were taken at 32 °C within 4 h after the cells were taken out from the incubator. According to our observations, the cells preserve their viability during this period. Prior each measurement the spring constant of cantilevers was determined using a combination of thermal noise and Sader methods, available within the driving software^35–37^.

### Force spectroscopy

Force maps were recorded with a resolution of 25 × 100 points at each selected area of usually 10 × 80 μm, total data collection time being less than 35 minutes. At every pixel of the map single force curves were recorded with a constant loading speed of 7 μm/s, using a piezo-extension rate of 1.2 Hz to minimize hydrodynamic and viscoelastic artefacts^8^. Total force distance was kept at 3 μm with a maximum load of 500 pN. The Young’s modulus was calculated from the approaching part of force curves, using a modified Hertz model^38^ based on the work of Sneddon^39^ and further developed for different AFM tip shapes^40–42^. The Poisson’s ratio of the cells was assumed to be 0.5, as suggested for soft incompressible materials^43^. All the force curves were fitted manually one by one in order to avoid any poorly fitted data points, as a possible consequence of automatic software calculations that may lead to an inaccurate elasticity map.

### Immunocytochemistry

Myotubes at 7 DIV in differentiation medium were fixed for 15 min in 4% paraformaldehyde in PBS, and incubated for 20 min in 15% donkey serum in PBS. They were then incubated 2 h at room temperature with the primary antibodies (anti-rabbit Actin 1:100, Sigma; and anti-mouse Myosin Heavy Chain 1:100, DSBH, A4.1025). After a wash in PBS, cultures were incubated for 1 h at room temperature with secondary antibodies, and were mounted in Mowiol. Images were collected using Zeiss 40X EC Plan Neofluar 1.3NA oil objective.

### Data analysis and statistics

The forces were analyzed within the Asylum Research software. Image processing was performed with ImageJ software. All data are reported as mean ± standard error of the mean (SEM). An F-test for equal variance and two samples t-test for means comparison (significantly different or not) were performed. Statistical significance was set at *p* ≤ 0.05.

### Data Availability

The datasets generated during and/or analysed during the current study are available from the corresponding author on reasonable request.

## Electronic supplementary material

Supplementary Information
