# Supplementary material for: Myotube elasticity of an amyotrophic lateral sclerosis mouse model
Source: Sci Rep. 2018 Apr 12;8:5917. doi: 10.1038/s41598-018-24027-5 (PMC5897453; doi:10.1038/s41598-018-24027-5)
Supplement: Supplementary file 1 — Supplementary Information [file 41598_2018_24027_MOESM1_ESM.pdf]

Myotube elasticity of an amyotrophic lateral sclerosis mouse model

Béla Varga, Marta Martin-Fernandez, Cécile Hilaire, Ana Sanchez-Vicente, Julie Areias, Céline Salsac, Frederic J.G. Cuisinier, Cedric Raoul, Frédérique Scamps and Csilla Gergely

Supplementary Information

1. Genotyping

Genotyping was carried out on DNA isolated from tail clips. A multiplex PCR with the following primers: SOD13 Forward: 5'-TTCTGTTCCCTTCTCACTGT-3'; SOD13 Reverse: 5'-TCCCCTTTGGCACTTGTATT-3'; SOD15 Forward: 5'-TGTTGGGAGGAGGTAGTGATTA-3'. SOD15 Reverse: 5'-AGCAGAGTTGTGTTAGTTTTAG-3'.

2. Quantification of gene expression

Myoblasts were seeded at the density of 10,000 cells per cm^2 in 60 mm collagen coated plates (280,000 myoblasts per plate). Total mRNA was extracted with the RNeasy Mini Kit (Qiagen) as described by Pieraut *et al.* [1]. The eluted mRNA was quantified by spectrophotometry (Nanodrop). Following gDNA wipe out, RT was performed with Quantitect RT kit (Qiagen). The primer sequences, designed with Primer-BLAST at NCBI, are listed in

Table **1**.

Table 1. **Primers**. Name, sequence, amplicon length (Al) and melting point (Tm) of primers used for qRT-PCR are listed below.

Target genes	Sequences	Al	Tm
Acta1<br>(Actin skeletal muscle variant 1)	F: AAGTCCTGCAAGTGAACAAGC<br>R: TTCGTCGCACATGGTGTCTA	80	59
Acta2<br>(Actin skeletal muscle variant 2)	F: ACGTGAAGCCTCACTTCCTA<br>R: TGTCTAGTTTCTGCTGCTCTG	64	58
Myh1<br>(adult Myosin Heavy Chain IIx)	F: TTCAAGTTTGGACCCACGGT<br>R: AGTGAGAGAGCCTGCCTTTA	54	59
Myh2<br>(adult Myosin Heavy Chain IIa)	F: TCCAAGTTCCGCAAGATCCA<br>R: GCGCATGACCAAAGGTTTCA	193	59
Myh3<br>(embryonic Myosin Heavy Chain)	F: TGTTGAGATTGCAGGATCTGG<br>R: TGCTGGGCTTTCCTGAACTT	121	59
Myh4<br>(adult Myosin Heavy Chain IIb)	F: TTCCGTAAGATCCAGCACGA<br>R: TCCTGTCACCTCTCAACAGA	151	58
Myh7<br>(adult Myosin Heavy Chain-beta)	F: TGAGCATTCTCCTGCTGTTTC<br>R: TGAGCCTTGGATTCTCAAACG	138	58

Myh8<br>(neonatal Myosin Heavy Chain)	F: ACAATCCAATGCCAACCTGG<br>R: TCCTTCCTCTGCAAGATGTGT	156	59
hSOD1<br>(human superoxide dismutase 1)	F: ACAAAGATGGTGTGGCCGAT<br>R: AACGACTTCCAGCGTTTCCT	162	59
Polr2j<br>(polymerase (RNA) II polypeptide J)	F: ACCACACTCTGGGGAACATC<br>R: CTCGCTGATGAGGTCTGTGA	160	59

Quantitative RT-PCR (qRT-PCR) was performed with 100 ng of total cDNA using SYBR Green (Qiagen) and the LightCycler system (Roche Diagnostics), after initial activation for 15 min at 95°C, 45 cycles of 94°C for 15 s, 60°C for 20 s and 72°C for 35 s were carried out. After PCR amplification, a melting curve analysis was carried out to ensure PCR specificity. Polymerase (RNA) II polypeptide J (Polr2J) levels were used to normalize the amounts of cDNA. ΔCt was calculated as the difference between the Ct values, determined with the equation 2–ΔCt.

3. Additional figures

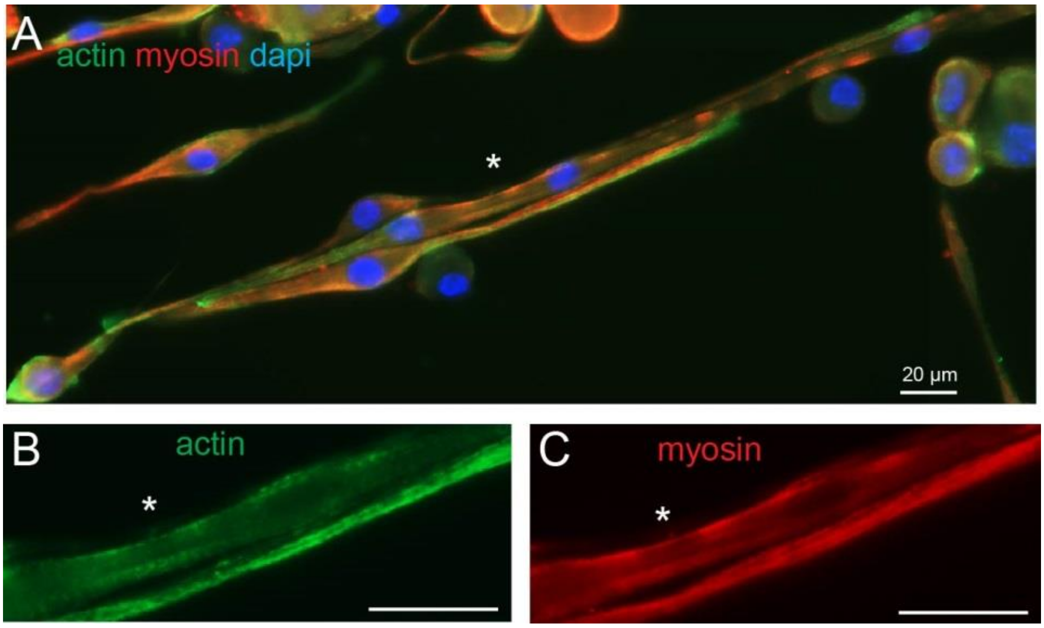

Figure S1. Maturation of wildtype myotubes. A: Immunofluorescence image of wildtype myotubes at 7 DIV with actin (green), myosin (red) and nucleus (blue) staining. B and C represent an enlargement of the marked area showing a more ordered actin (B), but a less ordered myosin distribution (C) along the myotube.

References

1. Pieraut, S. *et al.* An Autocrine Neuronal Interleukin-6 Loop Mediates Chloride Accumulation and NKCC1 Phosphorylation in Axotomized Sensory Neurons. *J. Neurosci.* **31,** 13516–13526 (2011).
